# Diabetic Ketoacidosis in Coronavirus Disease Patients With Type 2 Diabetes Mellitus

**DOI:** 10.7759/cureus.9731

**Published:** 2020-08-14

**Authors:** Soe P Winn, Zin Thawdar Oo, Nyein Nyein Htun, May Hnin Pwint Soe, May M Aung

**Affiliations:** 1 Internal Medicine, University of Medicine 1, Yangon, MMR; 2 Internal Medicine, LaSante Health Center, Brooklyn, USA; 3 Pathology, University of Medicine 1, Yangon, MMR

**Keywords:** covid-19, diabetes type 2, diabetic ketoacidosis, diabetes mellitus, corona virus disease 2019 (covid-19)

## Abstract

The occurrence of diabetes is increasing globally and carries a variety of complications, such as thromboembolism, acute cerebrovascular accidents, and diabetic ketoacidosis (DKA). Although DKA is not commonly associated with type 2 diabetes (T2D), it can manifest in patients who have underlying comorbidities predisposed to DKA. Since the emergence of the coronavirus disease (COVID-19) pandemic, we have seen many cases and studies on the underlying pathophysiology of the severe acute respiratory syndrome coronavirus 2 (SARS-CoV-2) pneumonia with or without respiratory failure. We have also learned that the angiotensin-converting enzyme receptor is one of the major entry sites of SARS-CoV-2 infection, and it might be one of the causes that predispose patients to DKA. However, few studies exist that explore the development of DKA in T2D with SARS-CoV-2 infection. We present two cases of patients with DKA and COVID-19 treated with an insulin regimen with no further complications.

## Introduction

Severe acute respiratory syndrome coronavirus 2 (SARS-CoV-2) infection has transformed into pandemic after the first case of coronavirus disease (COVID-19) was identified in December 2019 in Wuhan, China [1]. Information was limited regarding SARS-CoV-2 infection before the outbreak, and there were no treatment guidelines or vaccines for prevention. According to the US Centers for Disease Control and Prevention, COVID-19 poses a high risk of mortality and morbidity in specific groups of people, including those older than 65 years or people with underlying medical conditions like heart disease, lung disease, diabetes, and chronic kidney disease [2]. Diabetic ketoacidosis (DKA) is characterized by elevated blood glucose levels >250 mg/dL, arterial pH <7.30, and increased serum ketone level >3 mmol/L or significant ketonuria, which has been classically associated with uncontrolled type 1 diabetes [3]. It is also associated with type 2 diabetes (T2D) in African American patients, in a condition called ketosis-prone T2D or Flatbush diabetes [4]. During the COVID-19 global pandemic, we have seen increasing cases of COVID-19 patients with underlying diabetes presenting with DKA in non-African American patients. We report two cases of T2D with unusual presentation of DKA in patients infected with SARS-CoV-2.

## Case presentation

Case 1

**Figure 1:**
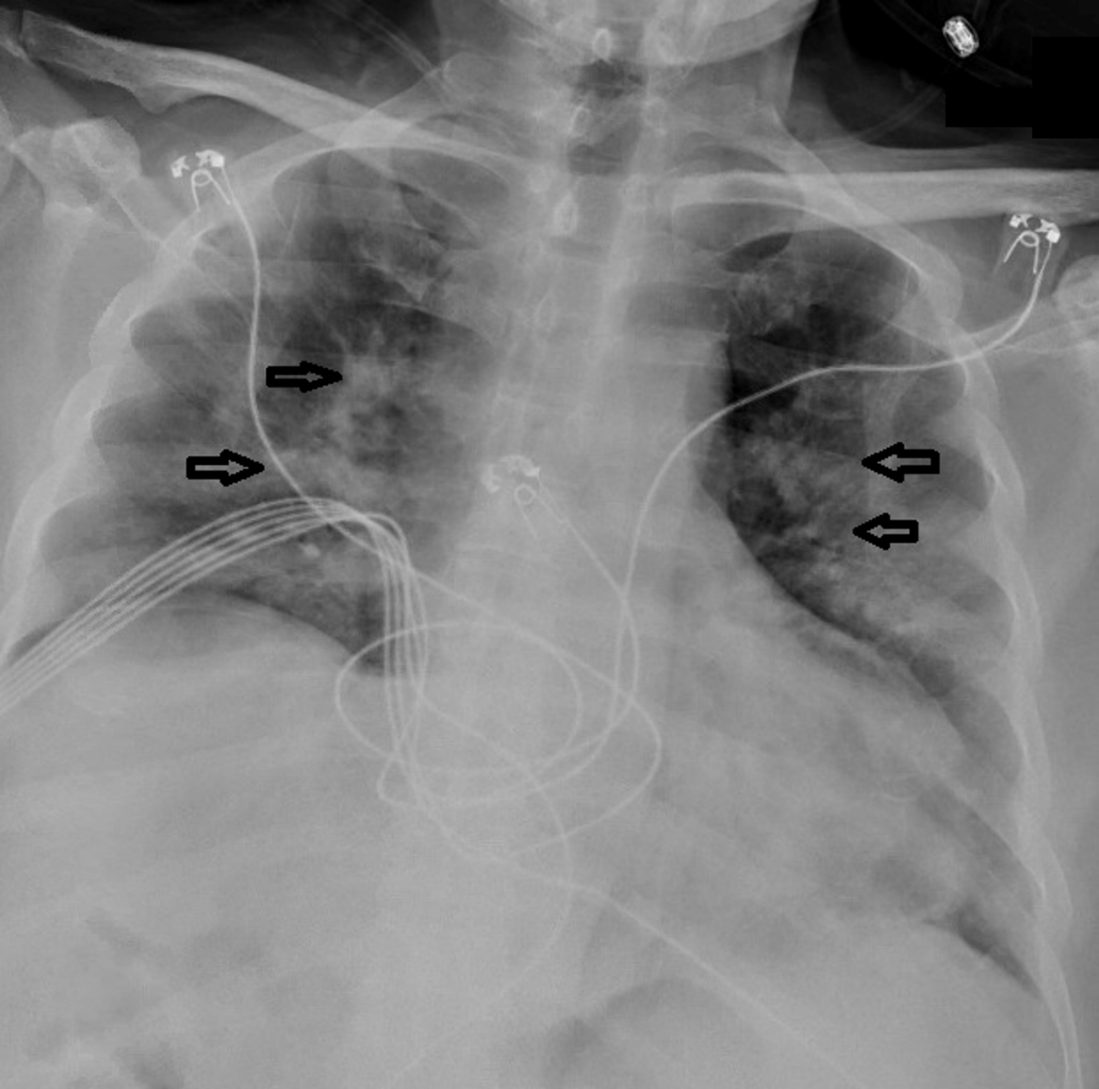
Portable x-ray (chest; AP view) showing bilateral pulmonary infiltrates compatible with COVID-19 pneumonitis. AP, anteroposterior

A 52-year-old Hispanic man with a body mass index of 25.7 kg/m^2^ and medical history of hypertension, T2D with neuropathy, and hyperlipidemia without previous exposure to glucocorticoid presented to the emergency department (ED) with mild fever, headache, and shortness lasting three days. His blood glucose levels had been well controlled with glipizide, metformin, and liraglutide, and his recent glycated hemoglobin was 7.2% (two months before presentation). On his arrival to the ED, his body temperature was 98.0°F, blood pressure was 130/82 mmHg, heart rate was 124/beats per minute, his respiratory rate was 34/breaths per minute, and peripheral capillary oxygen saturation (SpO_2_) was 96% on 10 L of high-flow nasal cannula (SpO_2_ was 88% on room air). His initial blood glucose level was 415 mg/dL with positive serum ketone of 5.8 mmol/L (reference range: 0.0-0.27 mmol/L) and urine ketone was 40 mg/dL (reference range: <0 mg/dL). His arterial blood gas analysis on 10 L of oxygen showed anion gap (AG) metabolic acidosis with pH 7.31, partial pressure of carbon dioxide (PCO_2_) of 27 mmol/L, partial pressure of oxygen (PO_2_) of 139 mmol/L, and bicarbonate (HCO_3_) of 13.9 mmol/L with elevated AG of 29 mmol/L (reference range: 8-12 mmol/L). D-dimer assay revealed 4,031 ng/mL (reference <318 ng/mL). Complete blood count showed elevated white blood cell counts of 23,100/µl (left shift neutrophil leukocytosis of 18,711/µl and neutrophil-to-lymphocyte ratio [NLR] of 13.5). His platelet counts, hemoglobin levels, and serum troponin levels were within reference ranges. Urinalysis showed no urinary tract infections. Inflammatory markers showed raised wide range C-reactive protein (CRP) levels at 14.122 mg/dL and increased ferritin at 1434.4 ng/mL (reference range: 3.1-110.9 ng/mL). Chest x-ray revealed bilateral pulmonary infiltrates compatible with COVID-19 pneumonitis (Figure 1), and the diagnosis was confirmed with a positive SARS-CoV-2 ribonucleic acid polymerase chain reaction (PCR) test.

He was admitted to the intensive care unit and treated with intravenous (IV) fluid and rapid-acting insulin infusion with closed monitoring of arterial blood gas for AG and serum sodium, chloride, bicarbonate, potassium magnesium, and phosphate every four hours. Every hour, we assessed his blood glucose levels and respiratory status for DKA, and we checked for respiratory distress. He was given broad-spectrum antibiotics (IV ceftriaxone 500 mg, twice daily, oral hydroxychloroquine 200 mg once daily, oral azithromycin 250 mg once daily), and supportive care for COVID-19 infection. His DKA resolved after 39 hours of insulin infusion with closed AG. We tested his serum C peptide level on the second day of his admission to assess endogenous insulin secretion, and it was low (0.6 ng/mL; reference range: 1.1-4.4 ng/mL) with a blood glucose of 193 mg/dL. His lipase level was within the reference range, indicating the patient was in an insulinogenic state. On the 21st day of hospital admission, he was discharged to home with the continuation of the same dose and regimen of oral antihyperglycemic medication (metformin extended-release, 1,000 mg orally, twice per day and glipizide, 5 mg, once per day) with advice to follow up with his primary care physician in two weeks.

Case 2

A 61-year-old Hispanic man with a medical history of T2D presented to the ED with fever, lethargy, cough, and diaphoresis lasting 15 days. He had stopped his diabetes medications and insulin for three days because of feelings of malaise and fatigue. His home oral antihyperglycemic agents are glipizide 5 mg, once per day and metformin extended-release 1,000 mg once per day. He has no previous history of hospitalization for diabetic complications, including DKA or surgery.

**Figure 2:**
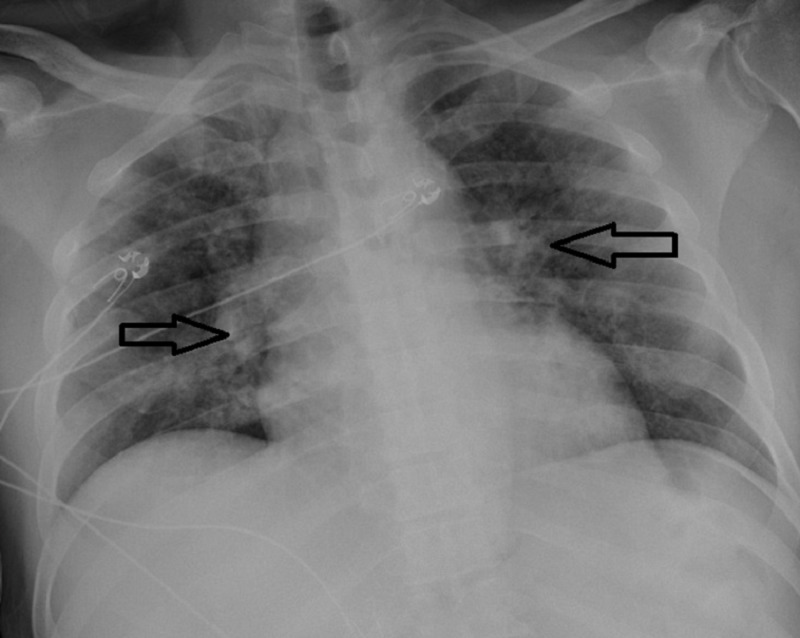
Portable x-ray (chest; AP view) showing bilateral pulmonary infiltrates compatible with COVID-19 pneumonitis (black arrows). AP, anteroposterior

**Figure 3:**
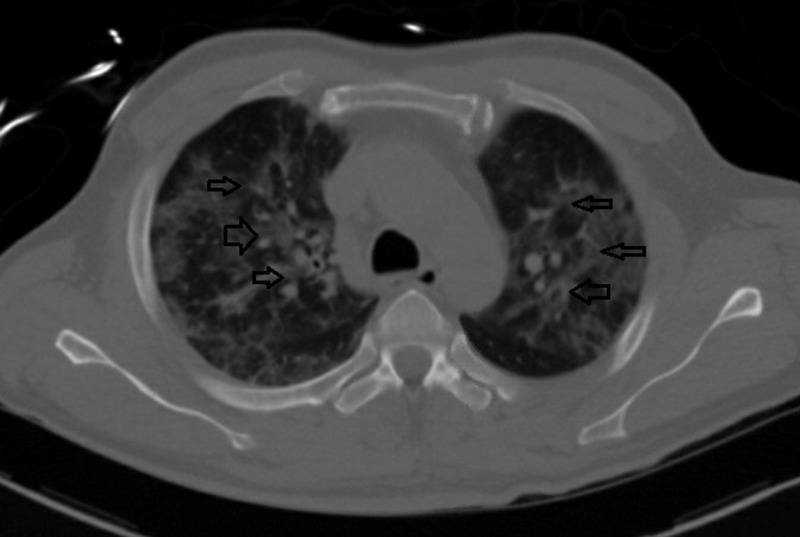
Chest CT shows diffuse bilateral ground-glass opacities/pneumonia compatible with COVID-19 infection (black arrows).

On presentation, his body temperature was 102°F, respiratory rate was 24 breaths per minute, heart rate was 96 beats per minute, blood pressure was 121/61 mmHg, and his SpO_2_ was 95% on room air. His initial blood glucose was 437 mg/dL with arterial blood gas showing AG of 16, with pH of 7.30, PCO_2_ of 27 mmol/L, PO_2_ of 139 mmol/L, and HCO_3_ of 15 mmol/L. Urine ketone was positive, and serum ketone was 4.8 mmol/L (reference range: 0.0-0.27 mmol/L). D-dimer assay revealed 3,680 ng/mL (reference <318 ng/mL). His complete blood count was significant for NLR of 12 with white blood cell counts of 9,700/µl. Both blood and urine cultures showed no growth. Chest x-ray and CT revealed diffuse bilateral ground-glass opacities/pneumonia compatible with COVID-19 (Figures 2, 3). This was later confirmed by a positive SARS-CoV-2 PCR test.

He was admitted to the hospital due to severe hypoxia with DKA and COVID-19 diagnosis. He was treated with high-flow nasal cannula 10 L/minute for hypoxia, subcutaneous insulin 20 U followed by IV rapid-acting insulin infusion (0.1 U/kg/hour) in normal saline briefly with hourly blood glucose monitoring until AG closure, fluid and electrolytes replacement for DKA, oral acetaminophen 650 mg every four hours as needed for fever, and subcutaneous enoxaparin 60 mg daily for deep vein thrombosis prophylaxis. The patient was discharged nine days after hospital admission with clinical improvement.

## Discussion

DKA is the major cause of morbidity and mortality in patients with diabetes. It usually results from a reduction of insulin levels in the body due to reduced secretion from beta cell units of the pancreas or increased insulin requirement due to stressors like sepsis. In one prospective observational study, the strongest risk factor for DKA development was precipitating infection compared with other risk factors, such as mismanaged insulin dosage, initial presentation, and other unknown causes [5].

COVID-19, like many other diseases, can predispose a patient to DKA due to the increased production of the stress hormone by stimulating cytokines. Interestingly, SARS-CoV-2 enters the body through inhalation of respiratory droplets by utilizing its envelope spike glycoprotein via binding to angiotensin-converting enzyme 2 (ACE2) receptor on human cell membranes [6]. Therefore, another possible mechanism for DKA development may be due to the deployment of ACE2 receptors by SARS-CoV-2 during their host-viral interaction, causing direct destruction and reduced function of pancreatic beta cells [7].

An animal-model study reported increased expression of ACE2 receptors in the pancreas of diabetic mice compared with non-diabetic, euglycemic mice [8]. Therefore, for patients with diabetes, it is reasonable to assume that the enhanced use of ACE2 receptors upon viral entry may cause receptor downregulation and increase the unopposed angiotensin-2-induced acute damage to pancreatic beta cells. Yang et al. also stated that the human pancreas also expresses ACE2, and therefore, patients with diabetes are more vulnerable to SARS-CoV-2 infection than the general population [9].

In our cases, the transient damage of pancreatic beta-cell function leading to reduced levels of serum C peptide may be the reason for our patients experiencing acute insulin-dependent DKA for a brief period during the course of COVID-19. In the respiratory system, ACE2 is necessary to break down angiotensin II to angiotensin 1-7, which are involved in the Mas receptor pathway to counteract inflammation and fibrosis. Therefore, a SARS-CoV-2 infection with the deployment of ACE2 yields a decline in the body’s defense mechanism alongside imbalanced metabolic function, leading to DKA and multiorgan dysfunction [6]. In the patients’ follow-up visits, they resumed their oral diabetic medications without insulin requirements. We did not perform follow-up C peptide level measurements because of the patients’ healthy fasting glucose levels managed with oral antihyperglycemic medications only.

## Conclusions

As illustrated by the these two patient cases, COVID-19 patients with T2D may present with unusual presentations of DKA. COVID-19 may cause DKA by increasing insulin requirement induced by ACE2-mediated destruction of pancreatic beta cells, as evidenced by reversible decreased serum C peptide levels or other unexplored mechanisms. Physicians should be aware of COVID-19 with a concomitant increase in the risk of DKA patients with T2D. However, more studies are needed for a better understanding of the pathogenesis of DKA in patients with coexisting T2D and COVID-19, and the prevention of poor outcomes in patients with diabetes.
